# Predictors based on cuproptosis closely related to angiogenesis predict colorectal cancer recurrence

**DOI:** 10.3389/fonc.2023.1322421

**Published:** 2024-01-09

**Authors:** Haoran Li, Yingru Zhang, Yuanyuan Feng, Xueqing Hu, Ling Bi, Huirong Zhu, Yan Wang

**Affiliations:** ^1^ Oncology Institute, Shuguang Hospital, Shanghai University of Traditional Chinese Medicine, Shanghai, China; ^2^ Department of Medical Oncology, Shuguang Hospital, Shanghai University of Traditional Chinese Medicine, Shanghai, China

**Keywords:** colorectal cancer, recurrence, prediction model, cuproptosis, prognosis

## Abstract

Up to one-third of colorectal cancer (CRC) patients experience recurrence after radical surgery, and it is still very difficult to assess and predict the risk of recurrence. Angiogenesis is the key factor of recurrence as metastasis of CRC is closely related to copper metabolism. Expression profiling by microarray from two datasets in Gene Expression Omnibus (GEO) was selected for quality control, genome annotation, normalization, etc. The identified angiogenesis-derived and cuproptosis-related Long non-coding RNAs (lncRNAs) and clinical data were screened and used as predictors to construct a Cox regression model. The stability of the model was evaluated, and a nomogram was drawn. The samples were divided into high-risk and low-risk groups according to the linear prediction of the model, and a Kaplan–Meier survival analysis was performed. In this study, a model was established to predict the postoperative recurrence of colon cancer, which exhibits a high prediction accuracy. Furthermore, the negative correlation between cuproptosis and angiogenesis was validated in colorectal cancer cell lines and the expression of lncRNAs *in vitro* was examined.

## 1 Introduction

Colorectal cancer (CRC) is the third most common cancer worldwide, accounting for nearly 10% of all cases (1). Although many react positively to treatment, including surgery, radiotherapy, and chemotherapy, up to one-third of patients experience postoperative recurrence with high mortality when the local tumor is completely controlled (2), and angiogenesis is one of the most critical factors for CRC progression.

Unrestricted invasive growth and metastasis of malignant tumors depend on angiogenesis, and tumors rarely metastasize without angiogenesis (3). When the tumor proliferates a critical amount, it starts the angiogenesis phenotype, produces strong angiogenesis activity, and enters the vascularization stage. New microvessels are the first step of tumor invasion and metastasis (4). The more tumor microvessels there are, the greater the chance of tumor cells entering the blood circulation. The wall of tumor neovascularization lacks structure integrity, in which there is only one layer of endothelial cells and lacks smooth muscle, which makes it easier to be penetrated by tumor cells than normal mature blood vessels (5). Furthermore, angiogenesis has a certain invasion ability, and tumor cells can invade along the collagen cracks attributed to vessels. Inhibition of angiogenesis can significantly suppress the growth of tumors (6). Anti-angiogenesis is a new strategy different from conventional anti-tumor therapy, and it has become a great prospect in tumor research.

In March 2022, Tsvetkov (7) proposed for the first time a copper (Cu)-dependent cell death mode, cuproptosis, which is different from other known cell death modes, such as apoptosis, necroptosis, and pyroptosis, it is a metal ion-induced regulatory cell death (8). Cu is an essential mineral for organisms and the basic element of many biological processes, including mitochondrial respiration, iron absorption, antioxidation, and detoxification. There is also some evidence that copper may play a role in the etiology, severity, and progress of cancer (9). Importantly, Cu can also promote angiogenesis, which is essential for tumor progression and metastasis (10). More and more evidence has shown that Cu can activate many angiogenic factors, such as angiopoietin (ANGPT), and vascular endothelial growth factor A (VEGFA) (11). It has been reported that Cu complexes and nanomaterials display the property of matrix metalloproteinase (MMP) inhibition (12, 13). It has been found that the Cu content in the serum and tumor tissue of cancer patients has changed significantly, which decreased in the serum of patients with CRC (14). Therefore, Cu-dependent cuproptosis may be related to the prognosis of patients.

Since the MOSAIC study (15), adjuvant chemotherapy has been a standard treatment for stage III colon cancer, which can prolong survival time and reduce the risk of recurrence. Only 20% of patients benefit from adjuvant chemotherapy, and 80% of the patients suffer unnecessary toxicity. Although clinical and pathological information is important in predicting prognosis, it is insufficient to determine which patients will benefit from adjuvant chemotherapy. Recently developed molecular markers, such as microsatellite status, BRAF, and KRAS mutations (16), which are instructional for immunotherapy and targeted therapy, are also expected to be important stratification factors for adjuvant chemotherapy. Furthermore, recent studies have emphasized the prognostic value of immune infiltration (17). Whether they are pathological, immunological, or molecular prognostic markers, these predictors can help clinicians stratify patients’ prognostic risks and develop individualized therapy.

Long non-coding RNA (lncRNA), a vast and unexplored region of the human genome, is a member of the non-protein coding RNA family with a length of more than 200 nucleotides. LncRNAs regulate the translation and decay of mRNA in a base-pairing-dependent manner (18) and participate in signal transduction through interaction with protein and lipids (19, 20). LncRNAs can affect signal pathways including WNT/β-catenin, PI3K/Akt, mTOR, and TP53 (21), and participate in many stages of tumor progression, including proliferation, apoptosis, angiogenesis, and metastasis. More and more transcriptome sequencing has identified many lncRNAs with altered expression and tissue specificity in cancer, which are expected to be potential prognostic markers. At present, most prognosis scores only use single-dimensional predictors: pathological data, immunity, or molecular markers. The analysis of large-scale multicenter clinical and molecular data can help integrate these factors into a comprehensive model. In this study, we aimed to verify the predictive ability of angiogenesis-derived cuproptosis-related molecular markers for colon cancer recurrence, and established a risk prediction model for colon cancer recurrence by combining clinical data with molecular biomarkers. This model stratifies the risk after radical resection, predicts the risk of recurrence, and is promising for guiding individual therapy.

## 2 Materials and methods

### 2.1 Collection and quality control of expression profiling by microarray

First, two microarray datasets based on GPL570 (Affymetrix Human Genome U133 Plus 2.0 Array) named GSE17536 and GSE17537 were selected from Gene Expression Omnibus (GEO) (22). The two datasets were obtained from expression profiling of colon cancer tissues in two medical centers. GSE17536 contains 177 samples, and GSE17537 contains 55. In this study, the original CEL files of microarray were selected for data analysis. The 232 microarrays were uniformly tested for quality, and the quality control was completed based on the R (version: 4.2.1) package “arrayQualityMetrics” (23), which includes five aspects: array comparison, array intensity distributions, variance mean dependence, Affymetrix specific plots, and individual array quality. Then, the RMA algorithm was used to sequentially perform background correcting, normalization, and summarization (24). RMA algorithm has performed logarithmic processing on gene expression. After the probes of GPL570 were annotated as gene symbols, the gene expression matrix was extracted. In the meantime, clinical information including gender, age, stage, outcome, and disease-free survival (DFS) time was further organized.

### 2.2 Identification of cuproptosis-related lncRNAs

mRNAs and lncRNAs were distinguished in the gene expression matrix through the annotation file of the UCSC Genome Browser (25). The mRNAs related to cuproptosis were determined through relevant published studies. Pearson’s correlation analysis was performed on cuproptosis-related mRNAs and lncRNAs using R, and the lncRNAs with linear correlation with cuproptosis-related mRNAs were identified as predictors preliminarily included in the model.

### 2.3 Constructing a prediction model based on the training set

GSE17536 was used as the training set for model construction and predictors screening, while GSE17537 was used as the validation set for subsequent external validation and predictive evaluation of the model. Event was selected as the outcome-related dependent variable, DFS was selected time as the time-related dependent variable, and sex, age, stage, and cuproptosis-related lncRNAs were selected as independent variables of the model. In the training set, the independent variables were tested using univariate Cox regression to evaluate whether they have a significant impact on survival to preliminarily screen the predictors. The screened predictors were used to construct a least absolute shrinkage and selection operator (LASSO)-based Cox regression model and further screened by stepwise selection to prevent the model from overfitting. The package “glmnet” (26) of R was used to construct the LASSO-based Cox regression model, which compresses the coefficients of predictors to 0 by setting the penalty coefficient λ, thus excluding the predictors that have few influences on dependent variables. LASSO regression selects λ with the smallest error in 10-fold cross-validations as the penalty coefficient. On the basis of LASSO regression, further screening was carried out by stepwise selection, which selects the best predictors by gradually deleting or adding predictors from the existing model and evaluating the prediction accuracy of the model.

### 2.4 Principal component analysis

Principal component analysis (PCA) is a method of inducing and combining multiple variables and distinguishing different samples with the least dimensions. PCA was carried out on the expression of all RNAs, cuproptosis-related mRNAs, cuproptosis-related lncRNAs, and the predictors to distinguish the ability of different biomarkers to predict risks. Meanwhile, scree plots were drawn to calculate the cumulative contribution rate (CCR).

### 2.5 Evaluation of model stability

The prediction model was constructed using the predictors screened twice and evaluated using influential point, multicollinearity, and the Schoenfeld individual test. The influential point was used to detect whether there was a sample that had a significant influence on the model fitting (that is, the sample had too much influence on the model compared with most samples), which would be eliminated. Multicollinearity refers to the significant correlation between the predictors in the model; that is, the same feature is described from two similar dimensions. This is due to inadequate screening of predictors and may result in model over-fitting. The Schoenfeld individual test was used to test whether there was a correlation between time and coefficient of the predictors, and if there was a correlation, the basic assumption of Cox regression was not established.

### 2.6 Internal validation and external validation

The number of predictors is greatly reduced after being screened twice, which may lead to the phenomenon of underfitting and decrease the prediction accuracy of the model. Therefore, the model was internally validated and evaluated for accuracy using the area under the curve (AUC), calibration curve, and Brier score. However, an independent dataset for external validation was selected, and the model was also evaluated using AUC, calibration curve, and Brier score. AUC and calibration curve are indicators to evaluate discrimination and calibration, respectively, and the Brier score was used to comprehensively reflect the discrimination and calibration.

### 2.7 Establishment of the nomogram

We drew a nomogram (27), which clearly manifested the prediction probability of the DFS of the samples. The nomogram scores the predictors according to their coefficients and variable types (classified variables or continuous variables) and outputs the survival probability according to the total score.

### 2.8 Risk division and survival analysis

The Risk Score (RS) was defined as the linear prediction of the model, and all samples were divided into high-risk and low-risk groups according to the RS median of the training set. Afterward, Kaplan–Meier (KM) survival analysis was carried out to explore whether there were differences in survival probability between high-risk and low-risk groups.

### 2.9 Enrichment analysis and immune cell infiltration

We performed Gene Set Enrichment Analysis (GSEA) on high-risk and low-risk groups identified using RS to verify the correlation between cuproptosis and angiogenesis. Subsequently, we performed Kyoto Encyclopedia of Genes and Genomes (KEGG) enrichment analysis and Gene Ontology (GO) enrichment analysis on cuproptosis-related genes to explore the signal pathway and biological function of their enrichment. CIBERSORT (28) quantified the composition of immune cells in tissues according to standardized gene expression data, and the accuracy of this method was verified by flow cytometry. CIBERSORT calculated *p* and root mean squared error for each sample, with a default signature matrix at 100 permutations, of which *p*-values <0.05 were filtered and selected for the next analysis. CIBERSORT demonstrated the difference in immune cell composition between the high-risk and low-risk groups.

### 2.10 Expression profile of colorectal cancer cell lines from public database

We selected all colorectal cancer cell lines in Depmap [Cancer Cell Line Encyclopedia (CCLE)] and downloaded the expression profile from Expression Public 23Q2, which was then normalized. After dividing the cell lines into two groups according to their sources, we carried out GSEA to find out the differences in angiogenesis and cuproptosis. Furthermore, we drew violin plots of cuproptosis–mRNA expression in colorectal cancer cell lines.

### 2.11 Cell lines and culture *in vitro*

The Caco-2 and SW620 cell lines were obtained from the cell bank of the Chinese Academy of Sciences (Shanghai, China). Caco-2 cells were cultured in high-glucose Dulbecco’s modified Eagle’s medium (DMEM; Gibco, Grand Island, NY, USA) supplemented with 10% fetal bovine serum (FBS; Gibco, Grand Island, NY, USA), 1% penicillin, and 1% streptomycin (Gibco, Grand Island, NY, USA) and supplemented with 5% carbon dioxide–air of a 37°C humidified incubator. SW620 cell line was cultured in Leibovitz’s L-15 (Corning, Shanghai, China) medium supplemented with 10% FBS, 1% penicillin, and 1% streptomycin.

### 2.12 Quantitative real-time PCR

Cell/Tissue Total RNA Isolation Kit V2 was used to remove genomic DNA and isolate total RNA. NanoDrop ND-1000 was used to quantify sample RNA, and III RT SuperMix for qPCR was used to further remove gDNA and perform reverse transcription. Real-time fluorescent quantitative was performed by ABI 7500 Instrument (Applied Biosystems, Foster City, CA, USA) using the SYBR Green method, and 2^−ΔΔCt^ was identified as the relative RNA expression. The target primers are shown in **Table 1**.

**Table 1:** Primer sequence.

mRNAs/lncRNAs	Primer
*MMP2*	Forward: TACAGGATCATTGGCTACACACC
	Reverse: GGTCACATCGCTCCAGACT
*MMP7*	Forward: GAGTGAGCTACAGTGGGAACA
	Reverse: CTATGACGCGGGAGTTTAACAT
*PDGFA*	Forward: GCAAGACCAGGACGGTCATTT
	Reverse: GGCACTTGACACTGCTCGT
*ANGPT2*	Forward: ACCCCACTGTTGCTAAAGAAGA
	Reverse: CCATCCTCACGTCGCTGAATA
*LINC02043*	Forward: GGAGCTCTCAGATGCTGGAC
	Reverse: CTACAGGGAGGTGGAATCCG
*LINC02754*	Forward: TTGGCAGGCTGGTATAAACTT
	Reverse: TGTGCTTGGTGGTGGTAATG
*LINC02510*	Forward: TTGGAATTGCCTGCTTTGAGC
	Reverse: CTCTGTTCTGGCAGGGTGAG
*DLEU1*	Forward: AGTGTTTGCCTTTACGCAGTC
	Reverse: GAAGCACTGCATGGTTGCAC
*GAPDH*	Forward: AATCAAGTGGGGCGATGCTG
	Reverse: GGGGCAGAGATGATGACCCT

## 3 Results

### 3.1 Quality control and annotation of microarray

**Figure 1:**
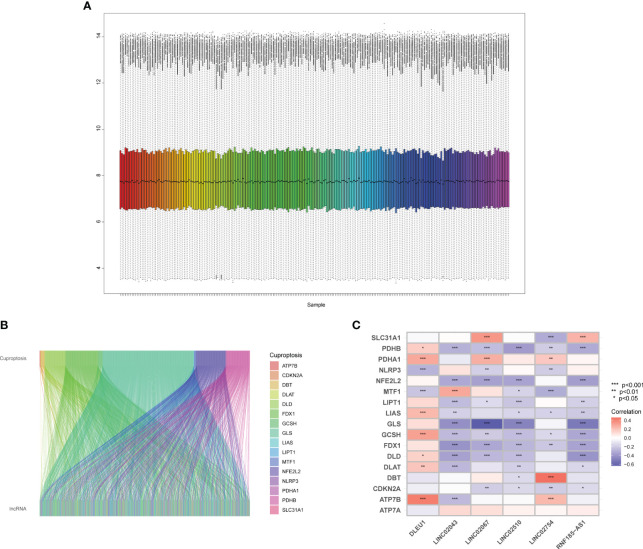
Quality control of microarray and identification of cuproptosis-related lncRNAs. **(A)** Boxplot of the expression profile of samples. **(B)** The lncRNAs have linear relationships with cuproptosis-related mRNAs. **(C)** Heatmap of cuproptosis-related mRNAs and predictors.

By analyzing the original CEL file, the grayscale (**Supplementary Figure S1A**) of the microarray is displayed, and a statistical analysis was performed to draw a barplot, boxplot, and MA plot (**Supplementary Figures S1B–D**), which shows the data distribution of the original array. Ideally, the scatter points in the plot are along the M = 0 axis. There may be problems with the microarray with a large interquartile range (IQR). After quality control, the microarrays with quality problems and without clinical data were eliminated. There were 145 samples in GSE17536 and 55 samples left in GSE17537. The probe matrix with 200 columns and 54,675 rows was normalized using the RMA algorithm, as shown in **Figure 1A**.

There were 54,675 probes in the GPL570. Since there were multiple probes detecting the same gene, we identified 17,202 mRNAs and 1,806 lncRNAs through the annotation file of the UCSC Genome Browser. There were 19 mRNAs (**Supplementary Table S1**) related to cuproptosis confirmed by relevant published studies, which were included in GeneCards—the human gene database (www.genecards.org) (29). Since the two mRNAs, *DLST* and *LIPT2*, were not annotated in GPL570, Pearson’s correlation analysis was performed on 17 mRNAs and 1,806 lncRNAs using R and detected 692 lncRNAs, which had a linear correlation (*p* < 0.001) with cuproptosis-related mRNAs (**Figure 1B**), among which there was no lncRNA significantly related to *ATP7A*.

### 3.2 Seven predictors after multiple screening

**Figure 2:**
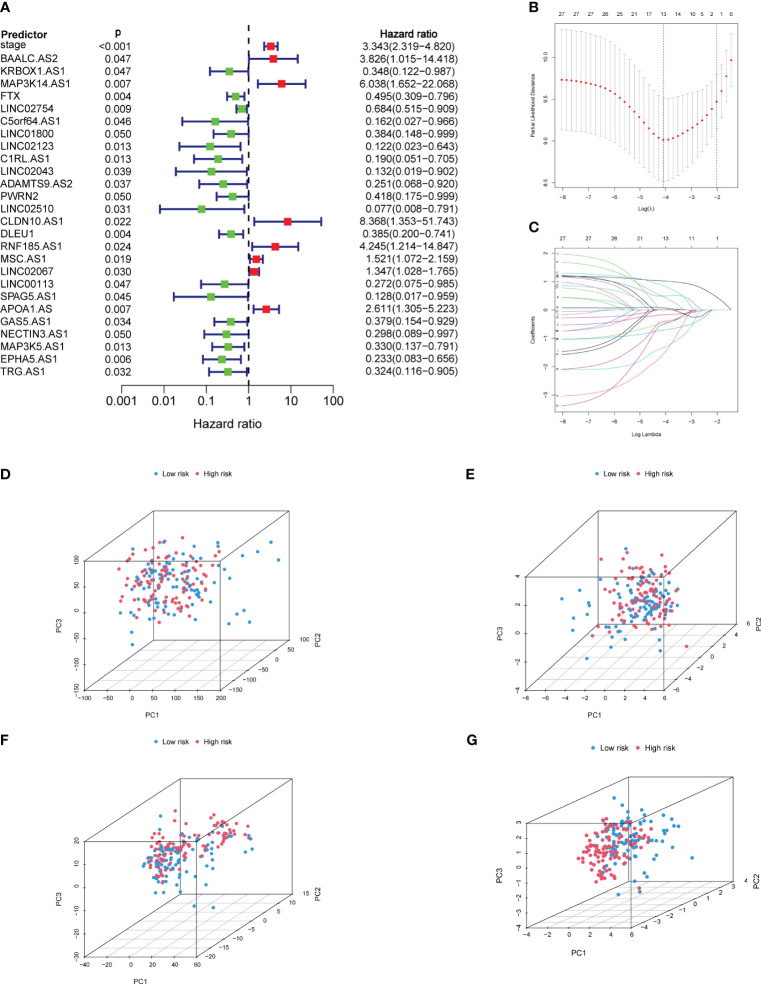
Screening of predictors and PCA. **(A)** Establishment of univariate Cox regression based on predictors. **(B)** The λ with the smallest cross-validation error in LASSO-based Cox regression was selected, and there were 13 predictors remaining in the model. **(C)** With the increase of the penalty coefficient, the predictors in the model gradually decrease. **(D)** PCA of all genes. **(E)** PCA of cuproptosis-related mRNAs. **(F)** PCA of cuproptosis-related lncRNAs. **(G)** PCA of predictors. PCA, principal component analysis; LASSO, least absolute shrinkage and selection operator. Red represents the high-risk group, and blue represents the low-risk group.

After the correlation test of univariate regression, 666 lncRNAs that had no significant influence on the outcome were excluded. Only “stage” and 26 lncRNAs were used as predictors for the multivariate regression (**Figure 2A**). In LASSO-based Cox regression, the event was selected as the outcome-related dependent variable and DFS time as the time-related dependent variable, nearly half of the predictors were screened out with λ = 0.243, and there were 13 predictors left in the model, including “stage” and 12 lncRNAs (**Figures 2B, C**). Through the stepwise selection, seven predictors were finally selected, including a classified variable “stage” and six continuous variables (*LINC02754*, *LINC02043*, *LINC02510*, *DLEU1*, *RNF185-AS1*, and *LINC02067*). The correlation with cuproptosis-related mRNAs is shown in **Figure 1C**. PCA (**Figures 2D–G**) manifested that the screening process was beneficial in distinguishing high- and low-risk groups. Scree plots (**Supplementary Figure S2**) intuitively indicate that the CCR of the top 3 principal components (PCs) gradually elevated with further screening. The CCRs based on all RNAs, cuproptosis-related mRNAs, cuproptosis-related lncRNAs, and the predictors were 30.5%, 45%, 55.6%, and 64.5%, respectively, which also suggest that the discrimination between the two groups based on only three dimensions was insufficient (<80%).

### 3.3 Model test and stability evaluation

**Figure 3:**
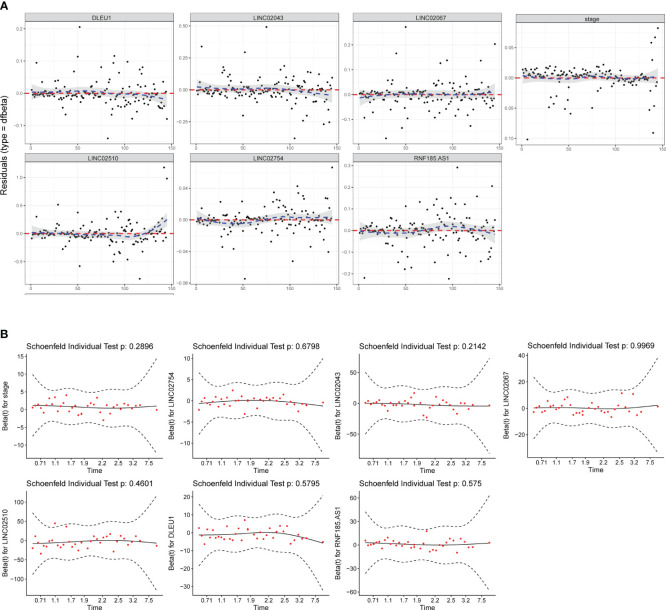
Model test and stability evaluation. **(A)** The influential point test is used to detect whether there are abnormal points with a strong influence on the model. The standardized residual for influential points is greater than 3. **(B)** The Schoenfeld individual test is to check whether predictor coefficients change over time. When *p* > 0.05, the proportional risk assumption of Cox regression is not rejected.

The test of influential point suggested that no obvious outlier was found in the residual diagram of predictors, and the residual of each predictor in the fitted model was close to 0 (**Figure 3A**). The multicollinearity analysis of package “rms” indicated that the variance inflation factors (VIFs) of each predictor were 1.083 (stage), 1.035 (*LINC02754*), 1.069 (*LINC02043*), 1.062 (*LINC02510*), 1.124 (*DLEU1*), 1.035 (*RNF185-AS1*), and 1.092 (*LINC02067*). When VIF < 5, it is considered that there is no multicollinearity between the predictors. In the Schoenfeld individual test (**Figure 3B**), *p* > 0.05 indicated that the proportional risk assumption was not rejected, and the coefficients of the seven predictors were not time-dependent.

### 3.4 Internal validation and external validation confirmed the high accuracy of the prediction model

**Figure 4:**
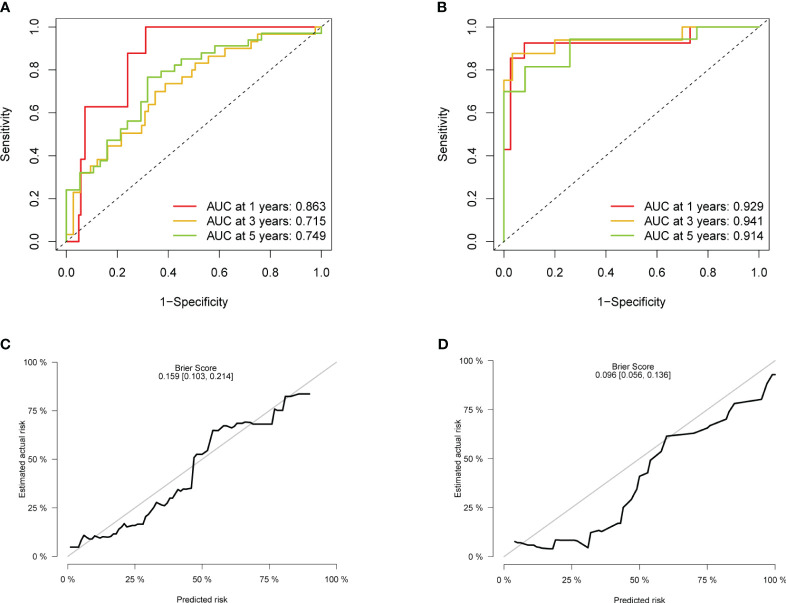
Evaluation of the accuracy. **(A)** ROC of the model in the training set. **(B)** ROC in the validation set. **(C)** Calibration plot in the training set. **(D)** Calibration plot in the validation set. ROC, receiver operating characteristic.

First, statistical tests from the training and validation sets revealed no differences in “gender” (*p* = 0.623) and “age” (*p* = 0.094) between the two sets. After internal validation, the model performed well in the self-prediction of the training set. The AUC values of 1 year, 3 years, and 5 years in the receiver operating characteristic (ROC) curve were 0.863, 0.715, and 0.749, respectively (**Figure 4A**). On the basis of external validation, the AUCs of 1 year, 3 years, and 5 years in the ROC curve were 0.929, 0.941, and 0.914, respectively (**Figure 4B**). The longer the time span, the lower the accuracy of prediction. However, the AUC suggested that the discrimination of the model did not obviously decline within 5 years. The 5-year calibration curves of the training set and the validation set were relatively fitted to the ideal curve, with Brier scores of 0.159 and 0.096, respectively (**Figures 4C, D**). Meanwhile, we tested the accuracy of the model when there was only one predictor, stage, in the model. The stage itself had good discrimination (**Supplementary Figures S3A, C**) but a poor calibration with a Brier score >0.2 (**Supplementary Figures S3B, D**).

### 3.5 The difference in survival probability between high-risk and low-risk groups

**Figure 5:**
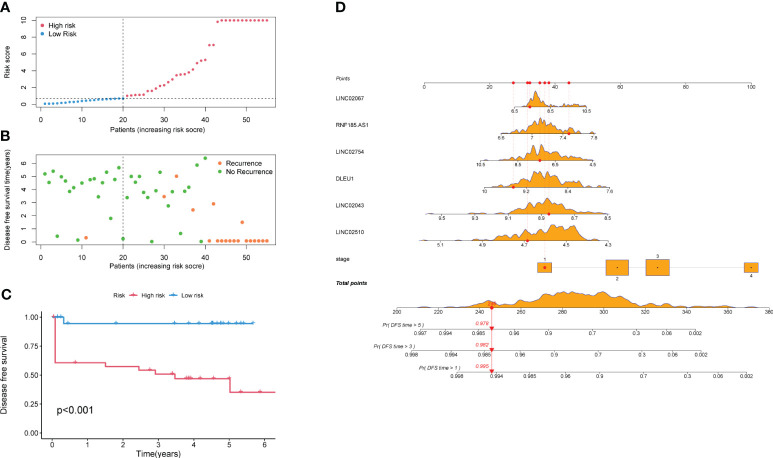
Survival analysis of high-risk and low-risk groups. **(A)** All samples are arranged by Risk Score. **(B)** Scatter plot of samples and their recurrence event. **(C)** KM survival curve. **(D)** Nomogram of the prediction model. The values of the seven predictors correspond to different scores, and the total score corresponds to the probability of DFS in 1, 3, and 5 years. The orange density plots show the distribution of training set data. The red lines represent the scores of predictors, total score, and corresponding survival probability, for the sample as an example. KM, Kaplan–Meier; DFS, disease-free survival.

All samples were calculated using the RS (**Supplementary Tables S2, S3**), which is the linear prediction based on the model, and the RS median of the training set was 0.822. The samples with an RS higher than 0.822 were classified as a high-risk group, and the rest samples were classified as a low-risk group. In addition, after sorting the validation set according to the RS (**Figure 5A**), it was found that the number of recurrences in the high-risk group was significantly higher than that in the low-risk group (**Figure 5B**). The KM survival curve (**Figure 5C**) indicated that the survival probability of the high-risk group was significantly lower than that of the low-risk group at the same time. The Logrank test suggested that there was a statistical difference (*p* < 0.001) in the distribution of survival time between the two groups. The nomogram intuitively revealed the predicted survival probability of samples in 1 year, 3 years, and 5 years (**Figure 5D**), as well as identified protective factors (*LINC02754*, *LINC02043*, *LINC02510*, and *DLEU1*) and risk factors (stage, *RNF185-AS1*, and *LINC02067*).

### 3.6 The differential expression of angiogenesis genes between high-risk and low-risk groups accompanied by different composition of immune cells

**Figure 6:**
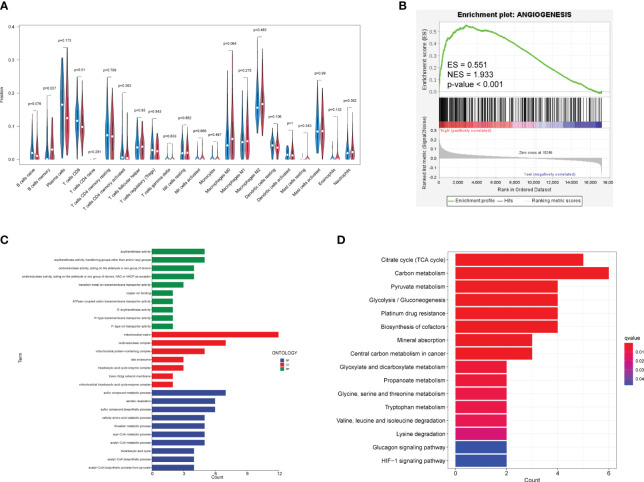
Relationship among immune infiltration, angiogenesis, and cuproptosis. **(A)** Violin plots demonstrate the difference in immune cells between high-risk and low-risk groups. Red represents the high-risk group, and blue represents the low-risk group. Nominal p-values are shown in the plot. **(B)** GSEA of two groups in the angiogenesis gene set. **(C)** GO enrichment analysis. Blue, red, and green represent the enrichment analysis of BP, MF, and CC respectively. **(D)** KEGG enrichment analysis. Red indicates significant enrichment. GO, Gene Ontology; BP, biological process; MF, molecular function; CC, cell component; KEGG, Kyoto Encyclopedia of Genes and Genomes; GSEA, Gene Set Enrichment Analysis.

The CIBERSORT analysis (**Figure 6A**) revealed significant differences in the composition of memory B cells (*p* = 0.027) and CD8^+^ T lymphocytes (*p* = 0.01) between the high-risk and low-risk groups. The proportion and the correlation of immune cells are also displayed in **Supplementary Figure S4**. Enrichment analysis (**Figures 6C, D**) showed that the cuproptosis-related genes refer to mitochondrial matrix processes such as acyltransferase activity, tricarboxylic acid cycle, acetyl–CoA metabolic process, acetyl–CoA biosynthetic process, pyruvate metabolism, mineral absorption, amino acid metabolism, carbon metabolism, and platinum resistance in cancer, which are not directly related to angiogenesis. However, GSEA demonstrated that the high-risk group defined by cuproptosis-related lncRNAs is significantly upregulated (enrichment score (ES) = 0.551, normalized enrichment score (NES) = 1.933, p-value <0.001) in the angiogenesis gene set (**Figure 6B**), suggesting the potential correlation between cuproptosis and angiogenesis.

### 3.7 Enrichment analysis of angiogenesis and cuproptosis in colorectal cancer cell lines and the expression of predictors *in vitro* validation

**Figure 7:**
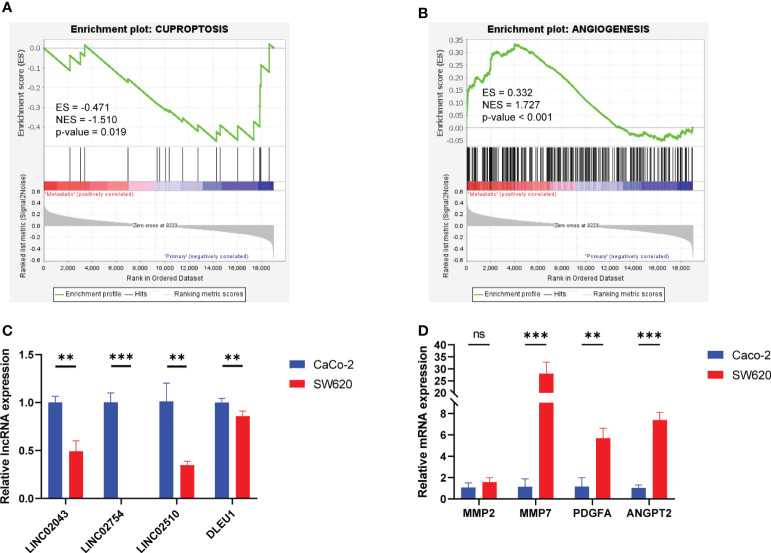
GSEA focusing on cuproptosis and angiogenesis of colorectal cancer cell lines and relative RNA expression of Caco-2 and SW620 detected by qPCR. **(A)** GSEA in the cuproptosis gene set. **(B)** GSEA in the angiogenesis gene set. **(C)** Angiogenesis–mRNA expression. **(D)** Cuproptosis–lncRNA expression. ns, *p* ≥ 0.05; **p < 0.01; ***p < 0.001. GSEA, Gene Set Enrichment Analysis.

CCLE recruited 56 primary cell lines and 20 metastatic cell lines. GSEA based on normalized expression profile (**Supplementary Table S4**) demonstrated that, relative to primary cell lines, metastatic cell lines were downregulated (ES = −0.471, NES = −1.510, p-value = 0.019) in cuproptosis gene set and upregulated (ES = 0.332, NES = 1.727, p-value <0.001) in angiogenesis gene set (**Figures 7A, B**). Violin plots exhibit expression of cuproptosis–mRNAs in different cell lines (**Supplementary Figure S5**). We cultured CaCo-2, primary colon carcinoma cell, and SW620, metastatic cell, and detected the expression of angiogenesis-related mRNAs and cuproptosis-related lncRNAs in the model by qPCR. It was found that the expression of angiogenesis-related mRNAs in SW620 upregulated significantly, while the expression of protective lncRNAs decreased significantly (**Figures 7C, D**).

## 4 Discussion

The prediction of tumor recurrence risk is of great significance for guiding prognosis and clinical decision-making of adjuvant therapy. At present, there are three scoring systems based on clinical data and pathological features: Memorial Sloan Kettering Cancer Center (MSKCC) score, ACCENT score, and Numeracy. The predictors include sex, age, carcinoembryonic antigen (CEA), histopathological grade, vascular invasion or lymphatic invasion, lymph node involvement, and adjuvant therapy. However, the prediction accuracy of the scoring systems was relatively low, with a c-index of no more than 0.7 (30, 31). MSKCC score (32) is a linear regression model (c-index = 0.68), which does not take time as a dependent variable, and can only be applied to stage II and stage III. Although Numeracy is a Cox regression model, the accuracy was insufficient (c-index = 0.65) in that it only included three predictors. ACCENT score (33), as a Cox regression model, does not use molecular markers as predictors, so its accuracy was insufficient, and it was only applicable to stage III patients.

However, the prediction model using a single biomarker is also quite defective. CEA, a carcinoembryonic antigen produced by gastrointestinal epithelial tumor cells, has been used as a tumor marker for colon cancer for more than 40 years. As a blood biomarker, CEA is an inexpensive, safe, and non-invasive measure for patients with colon cancer. However, CEA may be elevated for many reasons, including malignant and benign diseases, as well as smoking. Taken together, it is not an effective predictor of early clinical recurrence with a sensitivity of 0.5–0.8.

In this study, the prediction model based on biomarkers and clinical data innovatively integrated the two dimensions. Considering the stability of the Cox model, the recommended minimum events-per-variable (EPV) is 5–15 (34). Since the number of positive events in the training set was 36, the amounts of variables should be no more than 7.2. The pre-screening of potential variables by LASSO regression decreases the problem that the stepwise regression is less effective in the data with large variables. Compared with linear regression and logistic regression, Cox regression takes time as a dependent variable, which can predict the recurrence risk of samples at any time (35, 36). The AUC in the training set was higher than 0.7, the AUC in the validation set was higher than 0.9, and the calibration curve did not deviate from the ideal curve, which indicated a high accuracy of the model.

CD8^+^ T cells have the ability to detect and eradicate cancer cells. As shown in **Figure 6A**, there was a statistical difference in the proportion of CD8^+^ T cells between high- and low-risk groups, but the mean difference was small (approximately 1.56%). Compared with the difference between tumor and adjacent tissue, the difference in CD8^+^ T cells between high- and low-risk groups was relatively minor. However, risk stratification based on cuproptosis-related lncRNAs suggested a potential interaction between cuproptosis and the immune microenvironment, which also presents a prospect for prognosis prediction. Since the proportion of immune cells was calculated from the expression profile in this study, we could not use the immune infiltration as a predictor in reverse. We believe that the value of immune infiltration for prognosis is the detection of the various subtypes of CD8^+^ T cells by flow cytometry. In contrast, memory B cells were lower in the low-risk group. More and more evidence indicates that there is no inherent inhibitory effect on infiltrating B cells in tumors, and the induced regulatory B cells derived from the exposure to the tumor microenvironment, which plays an important role in inhibiting anti-tumor response and promoting tumor progress by weakening cytotoxic T lymphocytes and NK cells (37).

Relevant research indicates that *DLEU1*, as a protective predictor, is a candidate gene of tumor suppressor involved in B-cell chronic lymphocytic leukemia (38). *RNF185-AS1*, in contrast, has the effect of promoting proliferation and migration in thyroid carcinoma and liver cancer (39, 40). The effect of the two predictors in tumor progression confirmed their influence on the prediction of survival probability. As shown in **Figure 5D**, the higher the expression of *DLEU1*, the higher the survival probability, while the higher the expression of *RNF185-AS1*, the lower the survival probability. Similarly, although there is currently a lack of relevant research on other lncRNAs, we can speculate that *LINC02067* has the function of promoting tumor progression, while *LINC02754*, *LINC02043*, and *LINC02510* have the function of inhibiting tumor progression. This study is based on the widely recognized angiogenesis and new concepts of cuproptosis aiming to develop a more accurate prediction to evaluate the prognosis and recurrence of CRC patients.

However, microarray data derived from two different datasets must undergo unified normalization for comprehensive analysis, which increases the complexity of the study, and large-scale transcriptome sequencing studies should be carried out in the future to construct more adaptive models.

## 5 Conclusion

In this study, a prediction model for postoperative recurrence of CRC cancer was established, which combines clinical data and molecular markers with high prediction accuracy.

## Data availability statement

The datasets presented in this study can be found in online repositories. The names of the repository/repositories and accession number(s) can be found below: https://www.ncbi.nlm.nih.gov/geo/, GSE17536 https://www.ncbi.nlm.nih.gov/geo/, GSE17537.

## Author contributions

HL: Conceptualization, Data curation, Formal analysis, Investigation, Methodology, Resources, Software, Validation, Visualization, Writing – original draft. YZ: Data curation, Formal analysis, Funding acquisition, Investigation, Methodology, Resources, Software, Supervision, Validation, Writing – original draft. YF: Resources, Software, Writing – review & editing. XH: Supervision, Writing – review & editing. LB: Supervision, Writing – review & editing. HZ: Funding acquisition, Project administration, Supervision, Validation, Writing – review & editing. YW: Conceptualization, Funding acquisition, Project administration, Resources, Supervision, Validation, Writing – review & editing.

## Supplementary material

Supplementary Figure 1 Raw data of microarray. **(A)** The original gray scale of microarray. **(B)** MA-plot. Ideally, the scatter points in the plot are along the M = 0 axis. There may be problems with the microarray with a large IQR. IQR, interquartile range. **(C)** Histogram of signal intensity of probes. **(D)** Boxplot of the unnormalized expression profile of samples.

Supplementary Figure 2 PCA-related Scree plots demonstrate the contribution rate of principal components. **(A)** Scree plot of all genes. **(B)** Scree plot of cuproptosis-related mRNAs. **(C)** Scree plot of cuproptosis-related lncRNAs. **(D)** Scree plot of predictors. PCA, principal component analysis.

Supplementary Figure 3 Evaluation of the accuracy when there is only one predictor, stage. **(A)** ROC of the stage in the training set. **(B)** ROC in the validation set. **(C)** Calibration plot in the training set. **(D)** Calibration plot in the validation set. ROC, receiver operating characteristic.

Supplementary Figure 4 Immune infiltration by CIBERSORT. **(A)** Barplot indicates the proportion of different immune cells in each sample. **(B)** Correlation heatmap of 22 kinds of immune cells.

Supplementary Figure 5 Expression of cuproptosis-related mRNAs in colorectal cancer cell lines.
