# Supplementary figures and images for: Predictors based on cuproptosis closely related to angiogenesis predict colorectal cancer recurrence
Source: Front Oncol. 2024 Jan 9;13:1322421. doi: 10.3389/fonc.2023.1322421 (PMC10805227; doi:10.3389/fonc.2023.1322421)

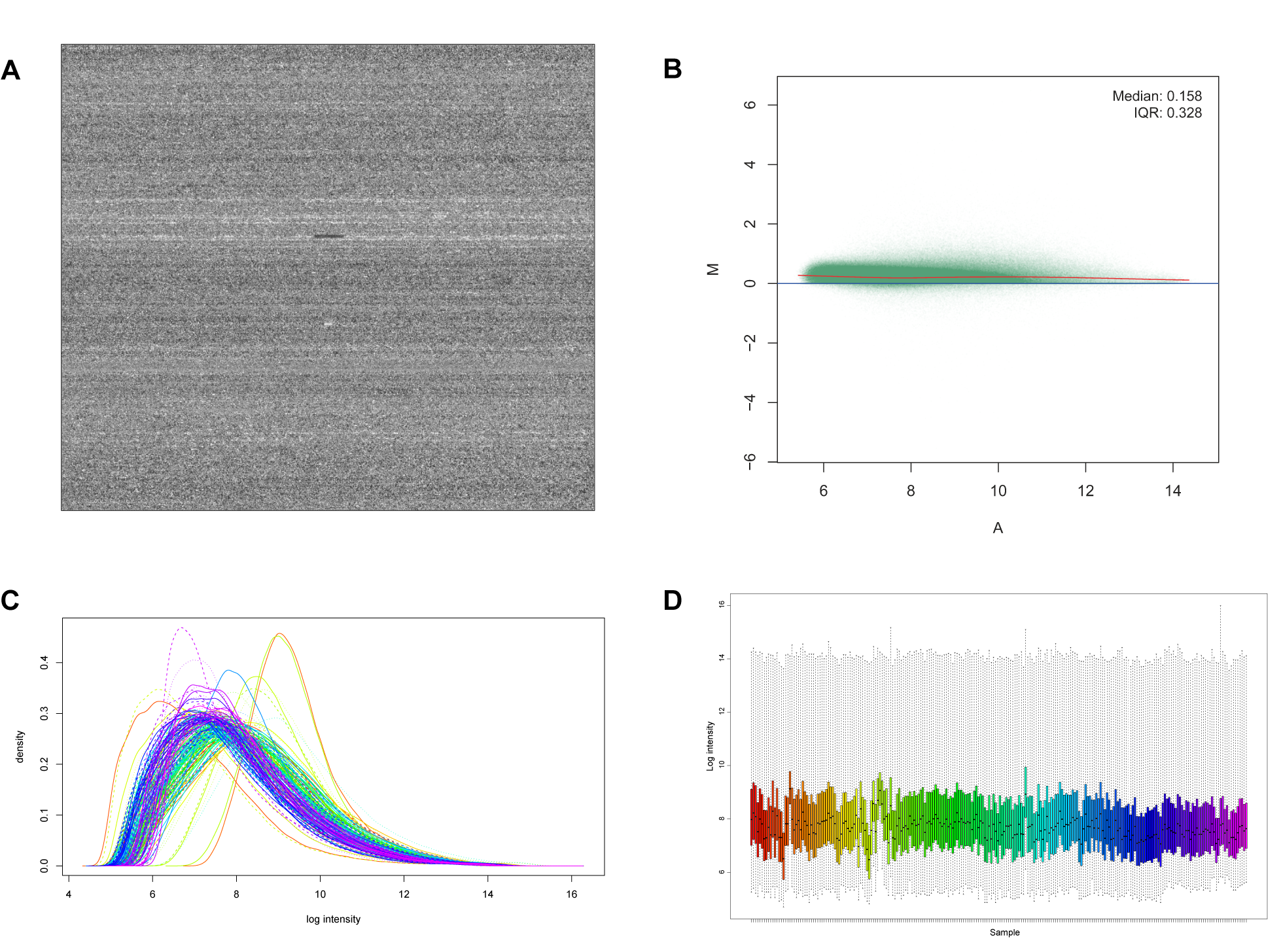

Supplement: Supplementary Figure 1 — Raw data of microarray. (A) The original gray scale of microarray. (B) MA-plot. Ideally, the scatter points in the plot are along the M = 0 axis. There may be problems with the microarray with a large IQR. IQR, interquartile range. (C) Histogram of signal intensity of probes. (D) Boxplot of the unnormalized expression profile of samples. [file Image_1.tif]

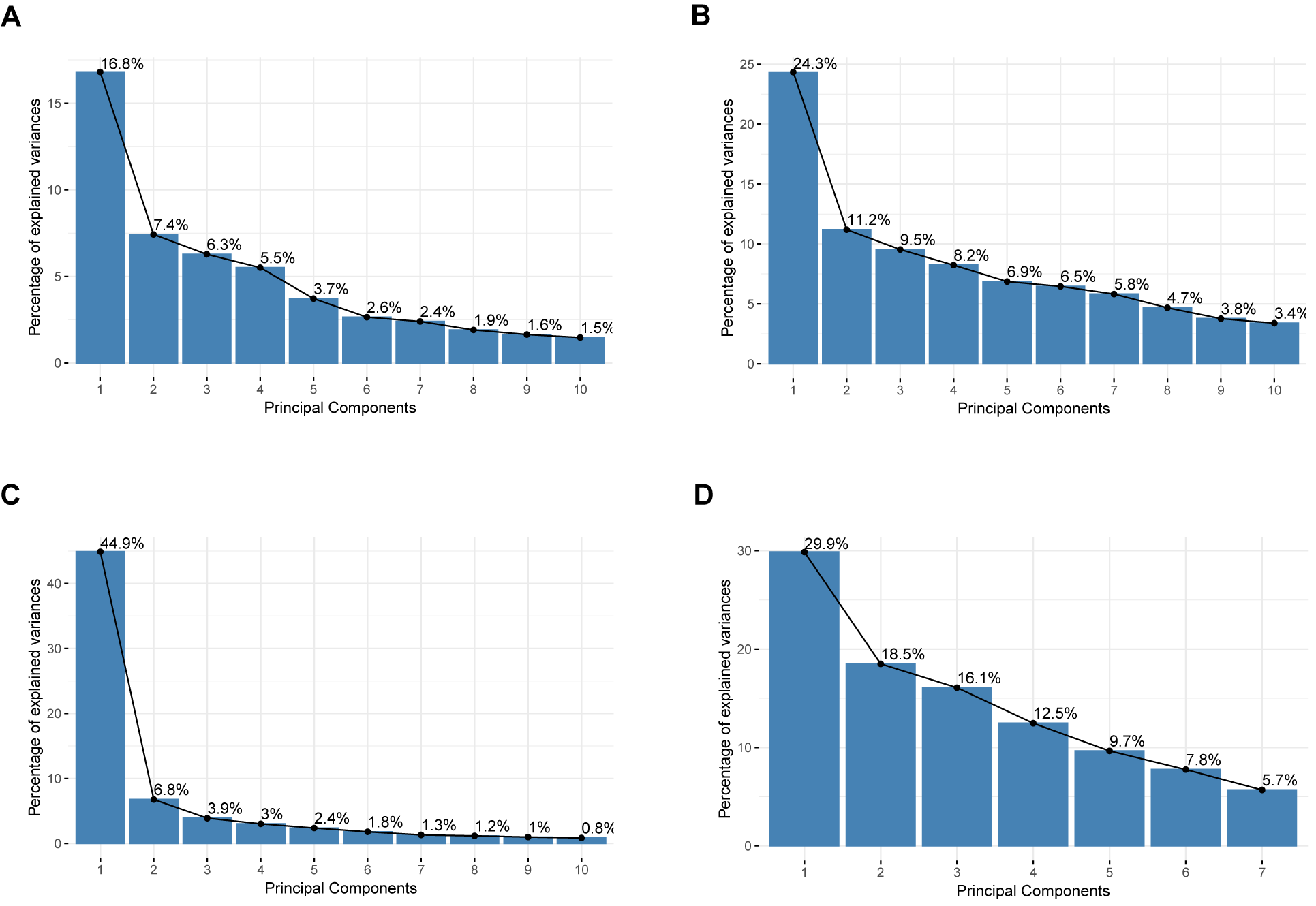

Supplement: Supplementary Figure 2 — PCA-related Scree plots demonstrate the contribution rate of principal components. (A) Scree plot of all genes. (B) Scree plot of cuproptosis-related mRNAs. (C) Scree plot of cuproptosis-related lncRNAs. (D) Scree plot of predictors. PCA, principal component analysis. [file Image_2.tif]

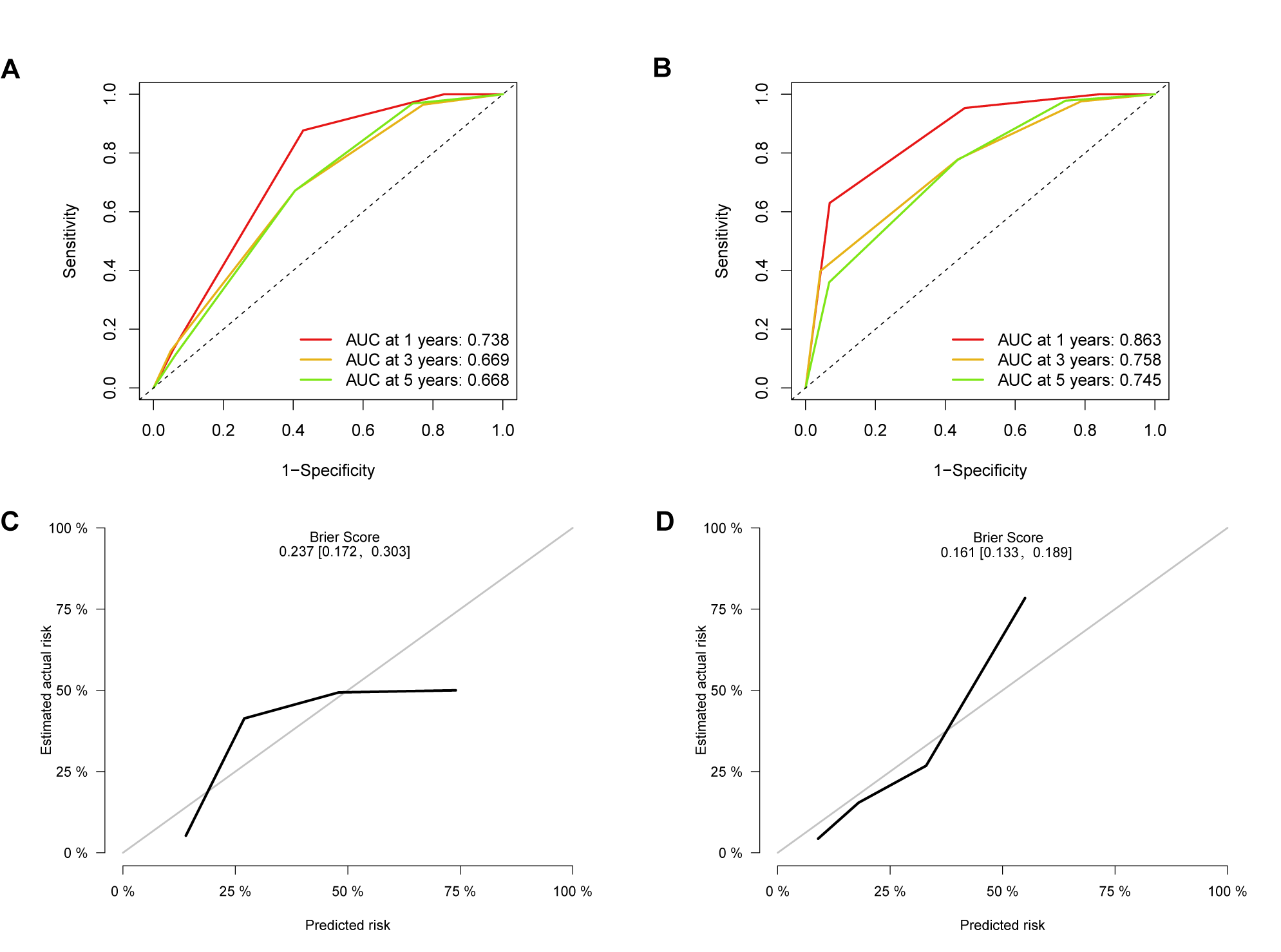

Supplement: Supplementary Figure 3 — Evaluation of the accuracy when there is only one predictor, stage. (A) ROC of the stage in the training set. (B) ROC in the validation set. (C) Calibration plot in the training set. (D) Calibration plot in the validation set. ROC, receiver operating characteristic. [file Image_3.tif]

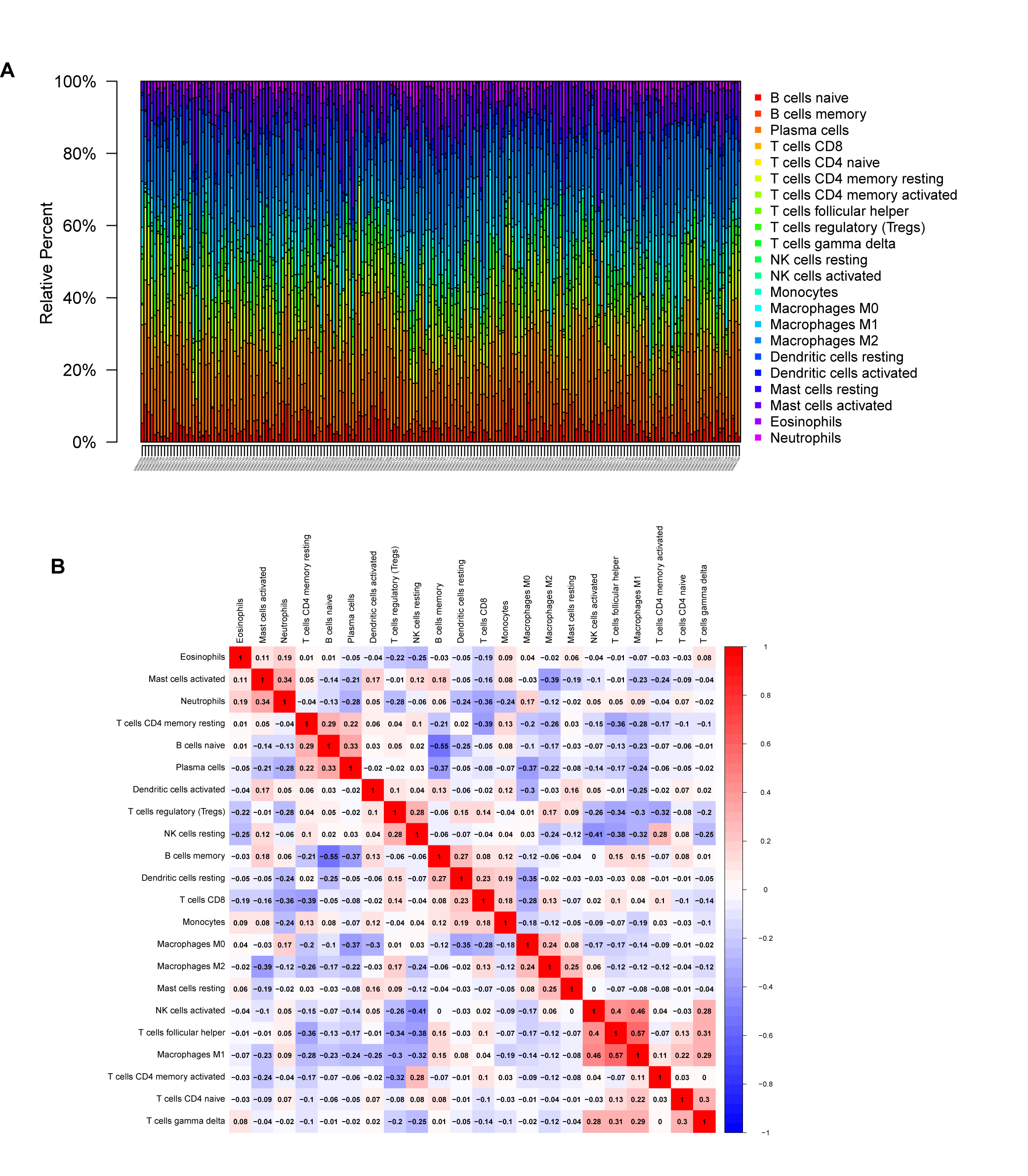

Supplement: Supplementary Figure 4 — Immune infiltration by CIBERSORT. (A) Barplot indicates the proportion of different immune cells in each sample. (B) Correlation heatmap of 22 kinds of immune cells. [file Image_4.tif]
